# Forced to Live: Controlled Forced Feeding of Political Prisoners and the Challenge to Nation‐States’ Civilising Processes

**DOI:** 10.1111/1468-4446.13196

**Published:** 2025-02-10

**Authors:** Stephen Vertigans, John Connolly, Paddy Dolan

**Affiliations:** ^1^ School of Applied Social Studies Robert Gordon University Aberdeen Scotland; ^2^ DCUBS Dublin City University Dublin Ireland; ^3^ Department of Social Sciences Technological University Dublin Dublin Ireland

**Keywords:** barbarism, civilising processes, Elias, forced feeding, hunger strikes, political prisoners

## Abstract

Since the nineteenth century, struggles between state power and political prisoners' right to die have aroused considerable interest. State enforcement to ‘make live’ through force‐feeding also raises important questions concerning processes that inform government approaches, often through methods considered to be brutal, and how these actions fit within perceptions of civilised behaviour. The social scientific focus of hunger strikes tends to be informed by Foucauldian bio‐power and governmentality which we draw upon when applying insights from figurational sociology. These insights allow us to better capture shifting social processes and changing public attitudes and behaviours that weaken state control over life and death. Different empirical examples are drawn upon, namely prison based forced feeding programmes that are directed at international ‘Islamicists’, Irish republicans and British suffragettes. Comparing groups' levels of integration within controlling states' societies, highlight distinctions in power balances, layers of mutual identification and entwined public perceptions and state reactions that help explain the implementation, cessation or continuation of force‐feeding.

## 1 Introduction

The practice of force‐feeding, what we collectively apply to political prisoners, who are on hunger strike, has been controversial since enforced on Russian revolutionaries during the nineteenth century (Grant 2011). Subsequent high‐profile cases include suffrage members of the British Women’s Social and Political Union (WSPU) early in the twentieth century, nationalist and ideological prisoners, including Irish republicans up to 1974, and then most recently post September 2001 ‘detainees’ or ‘enemy combatants of war’ in the American Guantanamo Bay. Analysis of these forced feedings have tended to focus on medical, legal, ethical and political dilemmas. The practices and reactions highlight tensions over states' responsibilities to look after their citizens and those in their ‘care’ alongside individual freedoms and their right to die (Clavan Powell 1983; Irmak 2015; Miller 2016; Scanlan, Cooper Stoll, and Lumm. 2008; Silver 2005). As Wilcox (2011) explains, tensions and acceptance of forced feeding often hinge upon psychiatric judgements about the extent to which the individual, usually a patient, is sufficiently rational to understand the consequences of refusing to eat.

The World Medical Association prohibited the use of force‐feeding in 1974 in cases where the prisoner is ‘capable of forming an unimpaired and rational judgment’ when choosing not to eat (cited in Ibrahim and Howarth 2019, 301). When the patient is classified as mentally deficient their liberal right to die is offset by the state's duty of care. In the UK, since 1994 prisoners have the legal right to refuse food and sustenance, providing they can demonstrate ‘sanity’ (Silver 2005). As this paper explains, America's approach is different with force‐feeding considered to be a prevention instrument that tackles hunger strikes as a form of suicide. In the case of Guantanamo, forced feeding fits within the government's proclaimed ‘ultimate responsibility that every detainee on our watch is taken care of’ (cited in Bargu 2016, 11).

## 2 Theoretical Framework

Sociological contributions to the debates about forced feeding have been heavily influenced by Michel Foucault (1978, 2003) and the struggle for control over the timing of death. Across Foucault's contributions, the role of state violence features heavily, stemming from Max Weber's (1978, 54) consideration of the ‘monopoly of the legitimate use of physical force in the enforcement of its order’. Such physical force is implemented within designated territory by a range of institutions including police, military and judiciary. Since Weber's exposition, sociological approaches to the monopoly of violence have explored how state institutions sought to legitimise and symbolise their use of violence and to project the neutrality of their instruments (Agamben 2005; Bauman 1989; Bourdieu 1991; Butler 2004; Gramsci 2011). The limitations of attempts at legitimation are exposed by people who choose to challenge and resist the monopoly. In the case of forced feeding as a form of state violence, this paper analyses both attempts to legitimise the implementation of forced‐feeding and challenges to the practice. Drawing upon the work of figurational sociology, most notably Norbert Elias, our framework builds upon power dynamics, politicised narratives and the targeted use of state instruments to illuminate these de/legitimising attempts (Dolan, Vertigans, and Connolly 2024).

Weber's contributions also informed Norbert Elias' exploration of the historical processes through which the monopoly of violence was established and extended by the centralising state (Kilminster 2014; Mennell 1998). For Elias, a centralising stable monopoly of violence (and taxation) was instrumental in shifting levels of interdependence and pacification within the civilising process. Impulses, desires and bodily functions were subjected to controls and rising levels of self‐restraints. Through the civilising process Elias sought to explain long term processes that helped shape the notion of Western consciousness and standards for behaviours and emotions that informed relationships and how people viewed themselves and others. The state’s monopoly of violence was not absolute, subject to challenge by competing groups, nations and individuals. The extent of this challenge would be informed by levels of pacification of behaviour and emotions, mutual identifications and repugnance concerning the use of violence (Fletcher 1997).

Application of the monopoly was to be confined to activities deemed threatening and in designated places, embedded within institutions, legal and bureaucratic processes that could be hidden from daily life. In so doing, the state sought to use violence to protect and maintain power. This paper concentrates on the tensions that surround the legitimacy of forced feeding as a form of violence and the challenge the practice can cause to notions of civilised behaviour.

Within contemporary states, the monopoly of violence is particularly evident within prison locations. Despite the apparent concentration of the monopoly within condensed, demarcated spatialities, prisoners retain agency, shaped through interdependencies and identification with non‐state groups, to contest the implementation of violence. The prisoners can resist in multiple ways such as breaches of etiquette through to disorder and rioting. Within these spaces, some prisoners challenge the state’s projected control over their lives by refusing to eat food.

State reactions to these prisoners’ claiming control over their life, have been captured within the concept of ‘bio‐power’ and the intent to ‘make live and let die’ (Foucault 2003, 241). For Foucault, institutional systems control and manage populations to internalise the requisite levels of constraint and compliance. Compared with Elias, Foucault placed greater emphasis on the development of this discipline through the mobilisation of different techniques within institutional settings such as schools, hospitals, prisons and factories. Physical, psychological and institutional domination and violence are control mechanisms for patients, pupils, workers and prisoners. Bio‐power is exercised through power and knowledge, the implementation of population management and public health measures.

A number of sociological contributions have applied these instruments of power and control to help our understanding into why hunger strikes are both practised and arouse significant public attention. Vicaro (2015, 183) captures the interrelation between bio‐power and hunger strikes when outlining how hunger strikers, ‘have seized this “weapon of the weak” as a means of persuasion designed to leverage the biopolitical state's obsessive concern with the bare bodily life of those in its control’. Bargu's (2016) adaption of Foucault and Agamben for hunger strikers in Turkey extends the focus to the politics of space and the foundation for asymmetrical conflict situations within institutions. Hunger strikers are, for Bargu, gaining control of their bodies in an act of ‘weaponization of life’ to resist institutional imposition of what Agamben (1998) described as ‘bare life’.

The resistance by hunger striking to government sovereign power within Foucauldian insights results in bio‐power as a ‘medical’ response to the prisoners' challenge. Forced‐feeding is directed at shaping the body and enforcing prisoners' dependence against their wishes (Melzer 2015; Purnell 2014; Wilcox 2011) through what Howland (2013, 102) describes as a ‘form of violent care’. Recognising the shift away from more subtle forms of disciplinary techniques, totalising regimes are believed to be challenging the hunger strikers attempt at sovereignty over their own lives. Through ‘medical’ discourse, knowledge, observation, monitoring and regulated procedures are applied that reinforces the power of the medical practitioners and the state's bio‐power over prisoners and life.

Howland (2013, 109) captures the extent of sovereignty and biopolitics when adapting Mbembe's ‘necropolitics’ to consider forced feeding to be ‘a politics of enforced life [original italics]—the state’s legitimate right to force individuals to live’. Bargu's (2016) concepts of bio‐sovereignty and necroresistance extend Foucault in the same direction of resistance to individualising and totalising power domination by state apparatus. For Bargu (2016, 85) ‘Necropolitical resistance transforms the body from a site of subjection to a site of insurgency …. Practices of necroresistance are thus both creative and destructive lines of flight that constantly escape being co‐opted into the biosovereign assemblage and destabilize the assemblage itself.’ Forced feeding does not feature in Bargu's study and we argue that the practice is an attempt by the state to regain control over the ‘escaping lines of flight’, lives and discipline. As Anderson (2010, 10) argues more generally, hunger striking ‘oscillate[s] in and out of control of the practitioner’.

The omnipresence of power and control is also integral to our study. However, the focus on Foucault's bio‐power, governmentality and controlling intent fails to adequately allow spaces for ‘bottom up’ resistance (Spierenburg 2013). Power and relations which inform opposition through the body need to be analysed to provide better insights into interconnections between life and power (Bargu 2016). Elias' relational positioning of power explicitly acknowledges different interconnected, often unintentional, dimensions. Longer term social processes are beyond institutions' spatialities and micro layers of subjugation, informing values, attitudes and behaviours with the potential to challenge the monopoly of violence. This extension of power dynamics can explain changes in approach and susceptibility both to shifting public consciousness which weaken state control over life and death and the ‘shifting balancing of tensions’ (Elias 1983, 145). Elias places greater emphasis on the changing and different social networks (figurations) that pressurise people into new ways of thinking, feeling and acting and their positions within these webs of interdependency (Dolan 2010; Burkitt 1994; Spierenburg 2013).

We argue that the different ways that people were subjected to forced feeding or similar treatments was not the mere result of new discourses or texts, ‘medical’ or otherwise, but the dynamic nature of the relations, particularly mutual dependences, between people. Of course, such changing relations often required new means of navigating such relations, which led to the production of texts or discourses, but pace the Foucauldian perspective these discourses were not the origin of such practice. Hence our figurational approach focuses more extensively on processes that contribute to government force‐feeding, public reactions and consequences for, or ‘barbaric’ challenge to, Western defined ‘self‐consciousness’ and the underpinning sense of being civilised. In so doing, we draw upon Elias's recognition of the duality of normative codes and tensions between safeguarding the nation‐state and/or the rights of the individual. We seek to understand how the implementations of forced feeding can reflect broader developments and patterns of interdependencies.

Three distinct categories of hunger striking prisoners, British suffragettes, Irish republicans up to the 1970s and Muslims incarcerated in Guantanamo are compared. Although prisoners' hunger strikes could be individualised, our examples are not isolated actions but take place within wider socio‐political programmes involving other participating prisoners and supporters. Reasons behind the hunger strikes include prison conditions, legal status, treatment and political injustice. These cases have been chosen because of their distinctions in terms of time periods, origins, ideologies, motivations and experiences. The 100‐year period of study captures the first suffragette hunger strike in 1909 and 21st century experiences in Guantanamo. These examples provide insights into the intersectional importance of gender, socio‐economic status, nationality, ethnicity and religion in attitudes for and against forced feeding and practices during challenging periods of British colonialism, postcolonial United Kingdom (UK) and the United States during the post 2001 phase of insecurity. Each category has contested their official classifications as ‘criminals’, in the first two cases, and ‘unlawful combatants’ in the case of Guantanamo where status has been much more legally ambiguous and hidden. Because of the contestation over their labels, treatment and politicisation of incarceration, the three categories are described in this paper as ‘political prisoners.’

Within analysis, consideration is given to how the implementation and ending of forced feeding intersects with other forms of consciousness and shifting established/outsider power dynamics and layers of mutual or we identifications around class, gender, ethnicity, religion and nation that inform ‘multiple hierarchies of integration’ (Wouters 2019, 169). Data is drawn from a wealth of archived and published interviews and auto/biographies of suffragette and republican prisoners subjected to forced feeding, prison diaries, security reports, media sources, histories and related academic contributions. Collectively the data includes differing accounts from hunger strikers, prison officials, members of legal and health professionals, politicians, reporters and academics. Through drawing upon a range of accounts, our paper takes ‘neither the dominant narratives of power nor the narratives subjugated by them at their word’ (Bargu 2013, 806). These multiple and varied sources allow for personalised and politicised accounts to be located within the shifting figurations and habitus that shape attitudes, responses to forced feeding, informed by colonial, postcolonial and gendered hierarchies. By comparison, the secrecy surrounding Guantanamo has resulted in less rich data and greater reliance on limited sources. Nevertheless, there is a sufficient range of resources to avoid overreliance on singular data while enabling the breadth required for comparative analysis. And Guantanamo is of relevance to our conceptual framework, depicted by sociologists as ‘the archetypical Foucauldian prison, an establishment where penal discourses, practices, and technologies are directed towards the bodies and mind of detainees’ Miller (2016, 10). We conclude by considering the indicators of de‐civilising processes such as levels of social‐constraints/self‐restraints, forms of mutual identifications, inter‐group fears and insecurities. In short, the paper explores how force‐feeding can help inform understanding about the shifting balance between normative codes and we/I balances before considering the implications for civilising processes.

## 3 Establishing Force‐Feeding

Force‐feeding is feeding against the individual’s wishes through the insertion of a tube into the mouth, nose, stomach, small intestine or anus. Many prisoners and detainees physically struggle and are then pinned down and violently restrained. How force‐feeding is described is informed by one’s sense of purpose. Opinions range from considering the practice to be torture, ‘artificial feeding’ and as a ‘medical procedure’. As the following analysis details, groups representing those being force fed will apply their discursive narrative in a manner that positions the practice as evidence in support of their wider goals.

Irrespective of ideological allegiance, hunger strikers have tended to share a similar sense of outrage, disgust and pain when describing being force fed. For instance, a Provisional Irish Republican Army (PIRA) hunger striker in 1973, Marion Price, described how,

Four male prison officers tie you into the chair so tightly with sheets you can’t struggle. You clench your teeth to try keep your mouth closed but they push a metal spring device around your jaw to prise it open. They force a wooden clamp with a hole in the middle into your mouth. Then they insert a big rubber tube down that. They hold your head back. You can’t move. They throw whatever they like into the food mixer … (cited in Miller 2016, 200).

When the feeding has finished, Laura Ainsworth, a suffragette in 1909, explains how,

a great deal of mucous and phlegm comes up with the tube. You feel quite stunned and dizzy and do a great deal of spitting for some time after the [18 inch] tube is withdrawn. You also have an ache in your chest and feel very sick (cited in Marlow 2015, 96).

In response to criticism of the practice, Reginald McKenna, British Home Secretary, declared in 1913 ‘If and when it [force‐feeding] causes pain, the pain is self‐inflicted by the prisoner’ (cited in Marlow 2015, 226).

In Guantanamo Bay, rolling hunger strikes started in 2002 (Velasquez‐Potts 2019). Force‐feeding in the military prison has been applied through nasal passage with prisoners handcuffed and restrained in feeding/restraint/torture chairs. This ‘restraint chair’, described by the manufacturer as ‘like a padded cell on wheels’ (cited in Wilcox 2011, 120), contributing to forced feeding being officially described as undertaken in a ‘humane and compassionate manner’ (cited in Ibrahim and Howarth 2019, 36).

This manner was challenged by Lakhar Boumediene, former prisoner (cited in Velasquez‐Potts 2019, 36) who described how,

you’re strapped into a six‐point restraint chair—we even call it the “torture chair”—and a lengthy tube is jammed into your nose and snaked down your throat. You feel as though you are choking, being strangled, and yet somehow still able to breathe. It’s an excruciating, impossible to describe feeling … at the same time, it is also torture to force a man to choose between giving up his only means of protest and giving up his life.

There are various reasons why political prisoners have been, and continue to be, forcibly fed. At one level the practice is a challenge to the purpose of the hunger strike. Governments decide whether the better outcome for them is to force the protesters to live or to allow them to die. This decision can be informed by anticipated public, prison and international reactions to the deaths of hunger strikers (Grant 2019; Jacobs 2012; Miller 2016).

The practice is also informed by preceding policies and models of medicalisation and control. Force‐feeding in Britain had been well established in prisons, hospitals and, most prominently for around 50 years, in asylums prior to the force‐feeding of suffragettes and Irish republicans (Geddes 2008). Shah (2022) details the Victorian phenomena of ‘fasting girls’ whose irregular or sparse eating habits mutated into anorexia nervosa. These ‘girls’ and young women could be institutionalised where they were force‐fed. In asylums such as Broadmoor, forced feeding of patients appears to have been informed by the medical discourse that continues to inform contemporary debates. During this period, the practice was considered a medical necessity for people incapable of rationally making decisions for themselves or whose illness prevented them from swallowing food through eating. By contrast with our examples, some of the patients were not necessarily on hunger strike but felt unable to eat food. Following these practices, mental instability and hysteria was frequently applied by medical practitioners and the media to suffragette hunger strikers in ways that suggested the WPSU members' behaviour was influenced by internal pathology rather than socio‐political critique. Hence, these ‘medical’ assurances were considered to justify the imposition of forced feeding despite the rigorous attempts by suffragettes to negate the associations with mental illness (Geddes 2007; Mackereth 2021; Shah 2022). The imposition of such restraints and interventions on females have been considered within practices of exercised total control over the body. In the process the body is changed from a site of resistance to one subjected to state biopower (Aretxaga 1995). While acknowledging the intended imposition of biopower our theoretical approach positions these sites of resistance within competing processes that seek to impose or challenge the justifications surrounding forced feeding.

## 4 Force‐Feeding as Barbarism

Following on from these experiences, force‐feeding as a practice has been positioned within a civilising and barbarism spectrum and as such fits neatly into sociological ways of thinking associated with Norbert Elias (Mennell and Dunning 1998; Van Krieken 1999) As is well documented, Elias outlined ‘civilising’ processes within Western Europe that have resulted in different levels of social and self‐restraints and the development of what became unconsciously accepted as modern ways of thinking and behaving (Elias 2000). Contrary to common misunderstandings, Elias did not position or judge levels of civilisation within a continuum of historical development. Instead, the sense of superiority held by some peoples who believe themselves and their culture/nation or religion to be ‘civilised,’ in contrast with barbarian others, is explored (Dunning and Hughes 2013; Mennell 1990; van Krieken 1998) and what Mennell (2007, 24) described as the ‘broad self‐approbation for “us Western people”’. However, as Elias (1996) explained within processes of civilisation, there can be contradictions and ambivalence. His example of the German Third Reich and the preceding processes that enabled the Nazis to gain power is a case in point. In this paper we are not arguing that the regimes under scrutiny are comparable to the Third Reich. Nevertheless, insights can be applied to the ambivalence surrounding actions and policies undertaken by self‐perceived civilised British and American governments and the challenge to their projected moral superiority. In our analysis we demonstrate how vocal, contemporaneous accusations of barbarity accompany shifts in balances of violence and pacification, self and social‐constraints and hierarchical forms of mutual identification. Consequently, examples are drawn upon that challenge the medical discourse and health rationale that position force‐feeding as acts of barbarism.

Across the different cases, medical discourse informs nation‐states’ basis for imposed feeding. For example, in 1909 the British Deputy Home Secretary, Charles Masterman, described suffragettes being fed by force as ‘ordinary medical treatment’ (Grant 2019, 61). This discourse was contested. For example, former Labour leader, Keir Hardie, denounced the ‘horrible, beastly outrage’, declaring ‘I was horrified at the levity displayed by a large section of Members of the House when the question [about force‐feeding] was being answered’ (cited in Pankhurst 1988, 317). Philip Snowden MP outlines the ‘method of barbarism’, which was a phrase adopted in 1901 to describe British military atrocities in South Africa. Slightly later, a suffragette newsletter also picks up the term, declaring that forcible feeding ‘is repugnant to all modern ideas of punishment and is a return to the dark ages of barbarism’ (Lenton in 1913 cited in Miller 2016, 49).

Irish suffragette striker, Hanna Sheehy Skeffington adopts sarcasm in 1912 when explaining that,

The hunger strike is a method of passive revolt that was initiated in Russian prisons where “politicals” adopt it where all else fails. In Russia they do not add the further refinement of cruelty—forcible feeding; it has been reserved to civilized England to adopt that method of “persuasion” (cited in Grant 2011, 141).

Following the escalation of WPSU protests to include actions such as the ‘argument of the broken pane of glass’ (Grant 2011, 135), there is limited evidence of either media (exceptions being the *Manchester Guardian* and, by 1914, the *Daily Mail*) or public protests against the practice of forced feeding. Popular attitudes were informed by beliefs that the political/criminal actions had been a threat to public order and private property (Vessey 2021). Moreover, legal cases brought against forced feeding were lost and the public ridiculed appeals on behalf of hunger strikers. These attitudes were consistent with broader opposition to the suffragette movement with protesters disrupting suffragette rallies, igniting sulphur, throwing objects at speakers and attacking marchers (Marlow 2015; Pankhurst 1988; Vessey 2021).

By contrast, the WPSU (and Irish republican groups) interpreted force‐feeding in part according to their overarching cause and opposition they faced. Suffragettes forced feeding was captured by Christabel Pankhurst in 1914 to be ‘all the barbarity, all the blind, brute force upon which the subject of women depends’ and the ‘violated bodies of women’, patriarchy and ‘this woman torturing Government’ (cited in Purvis 1995, 98). Rape was not explicitly mentioned in personal accounts of suffragettes. Nevertheless, the invasion of the body especially through the rectum or vagina was considered humiliating and an outrage (Purvis 1995; Shah 2022). Howlette (1996) points out during this period of history that ‘outrage’ was frequently applied in situations that would today be described as ‘rape’, ‘sexual abuse’ or Edwardian sexual violence (see also Aretxaga 1995; Purvis 1995). This association was identified within other later experiences such as women prisoners in the West German Red Army Faction who described forced feeding during the 1970s as ‘like rape. Each time I felt humiliated and destroyed’ (Margrit Schiller cited in Smith and Moncourt 2009, 259).

Rectal feeding suffragettes was believed to have largely been avoided due to ‘feminine delicacy and decorum’. Nevertheless, there were some reports of women being fed through the rectum and vagina (Pankhurst 1988; Purvis 1995; Shah 2022). News of the bodily invasions caused horror amongst the suffragettes and the stories clashed grotesquely with their promoted theme of sexual purity (Purvis 1995). That these accounts related to Perth prison in Scotland, where the facilities included an asylum, highlights variations in practice and ways in which the treatment for mental health patients continued to inform practices on prisoners (Pankhurst 1988).

The sexual abusive application of forced feeding has not been restricted to women. For example, within Guantanamo, Shah (2022) and Velasquez‐Potts (2019) note greater application of rectal feeding which they associate with sexuality, dominance and prisoner opposition to homosexuality. Implementing the practice was thought to further emphasise American masculinity and prisoner humiliation.

## 5 Multiple Hierarchies of Integration in Practice

When experiencing prison techniques, commitment to WPSU suffrage or Irish republicanism provided a sense of unity and friendship for hunger strikers. However, those experiences differed according to location and prisoner type. Methods of forced feeding were informed by multiple hierarchies of integration (Wouters 2019) such as gender, ethnicity, race and religion (Shah 2022). Alongside the obvious layer of gender, within the WPSU hunger strikers’ social class was also instrumental. Although the suffragette movement tended to be considered middle class, women from a range of working‐class occupations were reported within hunger strikers’ personal accounts (Marlow 2015; Pankhurst 1988; Purvis 1995). And within the prisoners, as Purvis (1995, 112) highlights, ‘their shared experiences were not experienced equally but fractured around a number of differences’. Such differences were partly informed by social habitus which led to expectations, requirements and adaptations to prison life being very different. And within the WPSU, there was recognition of the socio‐economic distinctions and opportunities that informed how the leadership aimed to help their lower class, helpless and oppressed ‘sister women’ (Mackereth 2021). Perhaps unintentionally these aims contributed to the marginalisation and objectification of working‐class members as victims (Schwartz 2019).

Deliberate emphasis on feminine attire was to also provide a visible marker between the layers of WPSU members. These distinctions were both to connect notions surrounding gender and to challenge the stereotypes of suffragette appearance which often relied on masculine characteristics. Experiences of hunger striking were to be influenced by these characteristics and how they informed treatments by medical practitioners. For instance, high ranking and well‐connected women could encounter ‘privileged’ treatment. Their presence in prison created dilemmas for medical doctors who felt some deference and as such were less inclined to coerce. Moreover, Grant (2011) argues, these women were believed to be more fragile than those from working classes. This was a period that continued to associate women of higher social status with feminine beauty, emotional sensitivities and inclinations to collapse into male arms or the ‘fainting couch’ when facing difficulties. Hence it is unsurprising that Mary Leigh, a working woman, was the first to be force fed (Purvis 1995). Working class suffragettes were also likely to be force fed for longer with pain inflicted by staff. With the WPSU reporting differential treatment, Lady Constance Lytton, a suffragette, tested the inconsistencies. Previously she had been imprisoned, was referred to as ‘your ladyship’ by the chaplain and on ‘medical grounds’ was released early. Three months later she went to prison under the pseudonym of Jane Warton. This time she received no health check prior to force‐feeding and inferior care. Lytton was to also highlight the contemptible ways in which her working‐class alter ego had been treated (Howlette 1996; Pankhurst 1988; Purvis 1995).

Medical unease and variations in implementation of procedures were captured by Herbert Smalley, Medical Inspector of Prisons, when referring, in 1912, to ‘the natural hesitation of the Medical Officer to use force towards the opposite sex, more especially in the case of persons many of whom are cultured and of refined habits’ (cited in Grant 2011, 137). Emphasis on gendered characteristics was also expounded by suffragettes who tended to define the hunger strike as a distinct feminine tactic of protest and ‘exploited the paternalistic sensibilities of humanitarianism, and its positioning of the white, British, female body as vulnerable to male violence’ (Mackereth 2021, 60). Against this backdrop, and concerned that medical professional reservations were interfering with forced procedures, in 1912 Mr Harman, an influential physician who considered the women to be ‘abnormal excitable individuals’ (cited in Williams 2008, 1), declared that as,

long as the law exists, which declares suicidal attempts a crime, so long must agree that forcible feeding is emphatically necessary for people who adopt starving … and it is certainly a necessary and ordinary treatment to preserve their health as any other means for irresponsible people (cited in Jacobs 2012, 288).

He goes on to recognise the importance of ‘resisting sentimental class clamour, and in maintaining the discipline of places which are, after all, for evildoers’ (ibid.).

The challenge that implementing forced feeding brought to the medical profession was partly because of their role at the forefront of government policies. By connecting into medicalisation, government policies have stressed care and saving lives against the projected irrational, often suicidal, actions of the prisoners. Today this care can include institutional algorithms, measurements of calorific intake, ideal body weights and calculations for triggering ‘involuntary medical treatment’ that exemplify for Shah (2022) that biopolitical practices in Guantanamo are more pervasive than twentieth century techniques.

Despite these techniques, medical doctors have struggled to reconcile the maintenance of life against a patient's will. The requirement to preserve health, political pressures and personal allegiances for and against prisoners' causes can complement or conflict with practitioners' habitus. Such a quandary is typical of what Sim (1990) describes as the usual dual loyalty of doctors to ethics of their profession and needs of their institution and nation‐state with lower ethical standards during times of crises. In the case of the suffragettes there was initially limited public support from male doctors to cease the practice. Significant changes within the attitudes of the profession became noticeable in 1912, three years after the introduction of force‐feeding for suffragettes, following a clinical study of the effects (Geddes 2008). Geddes (2008) highlighted different attitudes in the medical profession with doctors identifying as female being much quicker to express their opposition to the practice. These dilemmas have been exacerbated within 21st century American policy. The shift towards greater subordination of medical practice to the military that followed the Gulf and Iraq wars has been described by Miles (2013) as the new ‘military medical ethics.’ In Guantanamo, Shah (2022) reports on further medical and military divisions of labour within forced feeding as doctors and nurses have been tasked with supervision and enlisted sailors with medical expertise implemented the techniques. Shah also highlights that the number of prison's military medical staff was close to the approximate prisoner population of 140.

A counter argument to the role of the medical doctors could be their preference for implementing the pain of force‐feeding rather than witnessing the slow painful death by hunger. The medical profession is also informed by processes of prisoner stigmatisation and projected threats from their ‘patients’ which informed how ‘terrorists’ were treated.

Femininity amongst the ‘respectable classes’, described above, connected into the medical practitioners' habitus and informed British Home Office policy to ‘protect’ against self‐imposed death and how force‐feeding was applied to suffragettes. The medical practitioners shared with these suffragette members interconnections with colonialism and the overarching sense of British superiority and progressive civilisation. Such consciousness informed WPSU references to progress, moral values, the self‐preservation of race and support for Empire (Purvis 2013; Rendall 1994). Rendall (1994: 141) noted the suffragettes ‘consciousness of progress, of participating in a progressive movement of civilisation, to be differentiated from those other parts of the world still dominated by a “savage” brutality.’

Doctors more forcibly treated force‐feeding Irish republican hunger strikers, with whom they had much less in common and who were self‐declared opponents of the global civilising project. Moreover, republicans were considered physically and psychologically stronger, requiring less support which led to prisoner deaths (Miller 2016). As with the suffragettes' gender reinforcement, perceptions of masculinity were also emphasised by republican portraits of male hunger strikers and, with exceptions such as three female hunger strikers in the Armagh 1980 protest, women were excluded within wider portrayals of self‐sacrifice and ‘male heroics of violence’ (Lyness 2015, 158). Gender distinctions were also noticeable in the initial reluctance of the republican leadership to support the women because of ‘concerns that biological and psychological factors would make a woman's hunger strike more susceptible to defeat’ (Nugent 2016). Hence, both female and male symbolism, utilised by suffragettes and republicans, was to inform their levels of treatment.

Other relationships within prisons also had variable levels of empathy, indifference and anger. For instance, *Republican New*s (1976) provided an account of how prison staff behaved that was based on the experiences of Gerry Kelly and Hugh Feeney, who had been on hunger strike with Frank Stagg in 1974, ‘A team of screws are the first to appear. They come into the cell with varying expressions on their faces. These range from snarls, through passive indifference to the odd sheepish apologetic smile’ (cited in a republican source, *The Treason Felony Blog* 2018, 6).

Suffragette prisoners reported other experiences. The suffragette Kitty Marion described, in contrast to the violating male doctors, the sympathy of prison wardresses who ‘had fearfully tearstained faces … and I wished that the “brute things” who had ordered the f.f. could have suffered all these wardresses suffered, to say nothing of us’ (cited in Howlette 1996, 25). Sylvia Pankhurst (1988, 444) reported that these attitudes were more widely evident amongst ‘human beings who were torturing me came to the task with loathing and pity and would have refrained if they could’. Such perceptions were again not universally shared, and some suffragettes referred to the brutality and cruelty of wardresses towards them (Howlette 1996, 25). Following from the above hierarchies of treatment, Pankhurst's habitus and/or profile resulted in preferential treatment.

Relationships in Guantanamo have been shaped by the military regime and the treatment of ‘unlawful enemy combatants’, contributing to different interactions between wardens, medical staff and detainees (Howland 2013). For example, in 2005 after Army Colonel Mike Bumgarner, the military official in charge, agreed to hunger strikers’ requests, Major General Jay Hood overturned the decision. Hood’s primary role had been intelligence gathering and Vicaro (2015) argues that his lack of empathy with the prisoners and application of force‐feeding was informed by his focus on them as organic life‐forms. Rather than recognise the prisoners as human beings and political actors, Hood was believed to consider them as medical patients suffering from malnutrition that required intervention.

## 6 Stopping Force‐Feeding

Examining the processes into stopping or suspending force‐feeding provides valuable insights into how the strikers were considered and concomitant levels of support or opposition. Practical matters have also informed policies such as doctors' fears over being charged for manslaughter that led to early suffragette and Irish strikers being released after a short time of imprisonment. Officers have also been unable to cope with the disruption forced feeding causes prison order which also resulted in the early release of hunger strikers (Geddes 2008). When the number of hunger strikers grew and officials feared the tactic would be adopted by other categories of prisoners, the release tactic ended. However, greater awareness about forced feeding practices contributed to growing public opposition and dissent. As leading suffragette, Sylvia Pankhurst, reported ‘Even the *Daily Mail* protested: … “their application [of such force‐feeding tactics] to women … is barbarous and uncivilized. It converts a sentence of a month’s or two months’ imprisonment into a sentence of unbearable torment, degrading to the community which inflicts it”’ (1988: 453 [1931]).

Consequently, informed by both growing challenges to legitimacy and the burdens caused by hunger strikers on prison regimes, the British Government introduced the ‘cat and mouse’ approach in 1913 (Grant 2011; Purvis 1995). The new approach temporarily released hunger strikers, who were deemed eligible, until they were considered sufficiently replenished to be returned to prison. Ultimately, the WSPU's cessation of militant actions throughout World War I meant that imprisonment, hunger strikes and thus force‐feeding ended.

The Cat and Mouse Act was also implemented in pre‐independence Ireland where the death of hunger strikers was unacceptable to the British Government. Approaches to Irish republican force‐feeding were shaped by other pressures. The death of Thomas Ashe in 1917, after being mistakenly fed into his lungs, undermined the perpetuating argument that the practice was lifesaving. Instead, the case became evidence for opponents of torture and brutality against victims who were deemed powerless. Between 1917 and 1923 this pressure led to force‐feeding being stopped for republicans while continuing for first World War conscientious objectors and other types of prisoners.

In Britain, while members of the public did not sympathise with republican hunger strikers, opposition to forced feeding increased. Changing attitudes were partly informed by the shift from reform by punishment towards more rehabilitation and progressive treatments, widening forms of mutual interdependence and fluid forms of we identification. Hence, in the 1970s despite widespread opposition from the British population to PIRA, ‘torturing and degrading prisoners seemed to contradict deeply entrenched ideas on what it means to live in the modern, civilized West’ (Miller 2016, 193). Moreover, the role of medical paternalism was being challenged with the added dimension of force‐feeding republican female prisoners, notably the Price sisters who had been imprisoned in 1973. The 23‐ and 19‐year‐old siblings were characterised in some parts of the media in a manner that challenged preconceived ideas about PIRA activists and notions of republican masculinity. For instance, they were described as ‘girls’, which connected into the more widespread disconnect between gendered characteristics and political violence. Consequently, some accounts of the sisters' involvement described them as ‘victims of environment and background’. By partially describing the sisters as victims, these reports contributed to their force‐feeding being viewed to be a continuation of their earlier mistreatment. At the time there was also increasing association of force‐feeding with rape which connected to earlier narratives surrounding suffragettes (Howlette 1996; Purvis 1995; Shah 2022).

Highlighting how gender dynamics informed the force‐feeding debate, in 1974, two weeks after the Price sisters stopped being force fed, republican prisoner Michael Gaughan, died during hunger strike. Prior to his death, Gaughan's situation attracted little attention in comparison with the Price sisters. Following the uproar about the Price sisters and Gaughan's death, the British stopped force‐feeding prisoners. Then partly influenced by the British case, the World Medical Association declared force‐feeding to be unethical and ‘mentally competent’ people could no longer be fed against their will (although not legally binding). Moreover, force‐feeding had become interwoven with broader debates around human rights, civil liberties, torture and shifts in state dominance over individual rights (Miller 2016). Critics of force‐feeding were highlighting the levels of pain and suffering caused by force‐feeding and medical paternalism that were partly informed by the Nuremburg trials post Nazi bioethics developments in 1970s and 1980s that were to feature in West German opposition to force‐feeding of Rote Armee Fraktion (RAF) prisoners (Passmore 2009). Gradually the practice was to be challenged by the greater empathy for human suffering. Consequently, the argument that force‐feeding was government‐imposed pain developed throughout the twentieth century until it became too contradictory to notions of civilising behaviour.

## 7 Guantanamo: A Contemporary Exception

Within the UK and Irish examples, the growing public challenge in the 1970s to prison conditions and the decision to stop procedures was informed by concerns about rehabilitation approaches rather than the need for punitive measures including force‐feeding. In the USA the situation has been different with neo‐liberalism informing social‐constraints and greater punitive measures, that Simon (2007) has described as a waste management approach within models of incarceration.

Legally, force‐feeding continues to be disputed in the USA. Federal courts have recognised both the rights of individuals to die, and that force‐feeding may violate these rights. Nevertheless, judges have decided that the likely impact of death through hunger strikes on other prisoners and preservation of order takes precedence. And as Ibrahim and Howarth (2019, 302) report, a federal judge accepted the argument from lawyers representing Guantanamo prisoners that the procedure was a ‘painful, humiliating and degrading process’ that only the President could prohibit. Barack Obama, the president of the time, chose not to. Instead ‘enemy combatants’ continued to be stateless, denied national and international laws without many of rights granted to convicted criminals.

The legal situation is further compounded by variations according to American federal states and interests in preserving life, individual autonomy and privacy rights. Although attempts at suicide no longer result in criminal punishment, assisting someone does. This legality remains the case in the UK which has informed debates about the right to die most notably surrounding euthanasia. Moreover, common law systems have censured suicides for over 700 years and states often have a right to prevent suicide which exceeds individual rights (Clavan Powell 1983; Fessler 2003; Silver 2005). The US Supreme Court's 1990 ruling that,

As a general matter, the States—indeed all civilized nations—demonstrate their commitment to life … We do not think a State is required to stay neutral in the face of an informed and voluntary decision by a physically able adult to starve to death’ (cited in Fessler 2003, 244).

Following this application, hunger striking is legally a form of suicide and not the individual's right to die. Such a declaration is contested with Irmak (2015) capturing the essence of the counter argument that hunger strikes are not suicide because the primary intention is not to die. Velasquez‐Potts (2019, 27) explains, the hunger striker ‘does not desire death, but rather understands the possibility of dying as necessary risk inherent to political transformation’. Moreover, Bargu (2016) explains how individualising the action obfuscates the strikers' political messages. Establishing whether there is suicidal intent is important because applying the suicide narrative has enabled governments to portray themselves as providing a ‘duty of care’ that does not permit the right to die under these circumstances. As the above analysis highlights the notion of care had been applied throughout the twentieth century UK and Irish examples.

With regards to Guantanamo, the United States Defence Department declared that ‘preservation of life through lawful, clinically appropriate means is a responsible and prudent measure for the safety and well‐being of detainees’ (cited in Howland 2013, 107). Curiously when three detainees were contentiously reported to have killed themselves by hanging, their actions were detached from the suicide narrative when described by Harry Harris Jr, the Base Commander, as ‘not an act of desperation but an act of asymmetric warfare against us’. In other attempts at justifying force‐feeding, the American military has reinforced the medical model. Former Commander of the Hospital at Guantanamo, Captain John Edmondson, when defending his decision to force feed, declared that ‘I will not allow them to harm themselves’ (cited to Wilcox 2011, 101). Instead, medical practitioners were, ‘providing nutritional supplements on a voluntary basis to detainees who wish to protest their confinement by not taking oral nourishment’ (cited in Vicaro 2015, 181). On leaving the role, Edmondson was awarded a medal for his ‘inspiring leadership and exemplary performance [which] significantly improved the quality of health care for Guatanamo Bay’ (cited in Nicholl 2006). Yet a 2006 United Nations Human Rights Commission investigation into the prison regime upheld allegations of torture by excessive force when ‘providing nutritional supplements.’

The American approach can be located within a continuum of governments' justifications, positioning state institutions in the role of saviour. Such an approach is riddled with contradictions, as Howland (2013, 107–8) highlights in ‘a form of violent care where the preservation of the welfare and life of the prisoner is paradoxically pursued to the point of violence, temporary or permanent debilitation and even death’. Hence the practice of force‐feeding in Guantanamo is embedded within a period when the rehabilitation model is weaker, underpinned by differing American attitudes to rights, life and morality.

The importance of morality within American policies has a long‐standing history that has been applied to processes of stigmatising outsiders including the Guantanamo prisoners. Mennell (2007) explains how the moralising tradition has contributed to common classifications of who is ’bad’ from the days of the frontiers through to the treatment of prisoners in Abu Ghraib and Guantanamo. The moralised demonising of ‘enemy combatants’ has been formed within figurations that only weakly incorporate Muslims and lack insights into common history, experiences and knowledge of other regions and peoples. Large swathes of the American population uncritically accepted Muslims to be the savages in the struggle between ‘civilisation and barbarism’ (G. H. Bush, cited in Linklater 2021, 34). We‐forms of identification were rigidly based on exaggerated stigmatised characteristics of outsider threats and the determining status of the American established that Elias and Scotson (1965) outlined in their established/outsider analysis of community relations. In their study, the unequal power relations between the two groups and the established’ greater cohesion around historical memories, recent experiences and control over communication channels enabled them to shape their own image based on an unrepresentative selection of the ‘best’ members. Outsiders were portrayed according to the ‘minority of their worse’ representatives. Moreover, as Linklater (2021) explains in relation to the post September 2001 American use of torture, the discourse surrounding national security, global order and law principles became dominated by a willingness to shift the moral and legal boundaries. The reconfigured normative codes allowed overt forms of violent and constraining behaviour towards unrepresented individuals that would previously have been at odds within the projected sense, and self‐protection, of the ‘civilised’ nation‐state.

In the United States, Islamophobia and the fear of the other during times of crisis are also mobilised through diverse forms of communication interwoven with a national sense of cohesion in support of the physical isolation of outsider enemies with whom many Americans felt very little in common (Vertigans 2013). The significance of separating the established and outsiders is exemplified by de Swaan's (2001, 268) analysis that barbarism was compartmentalised within Nazi Germany through ‘demarcated spaces, in delineated episodes, well separated from the rest of society and the everyday existence of the other citizens.’ This separation was to enhance surveillance and social control while restricting processes of mutual identification (Mennell 1990). Similar processes occurred with the physical and social isolationism imposed in Guantanamo. The American population's overall willingness to allocate greater precedence to the protection of the nation‐state and the projected threat to the self‐conscious ‘civilised’ way of life compromised the normative code that respects the rights of all individuals.

## 8 Conclusion

Across the three cases, the counter arguments of the civilising sense of care and barbaric experiences of violence are striking. Foucauldians have highlighted tensions surrounding force‐feeding through the bio‐power, medical discourse that positions the practice as preventing self‐harm and saving lives. For hunger strikers there is no right to die. By comparison, oppositional denouncements consider acts of forced feeding to exemplify the brutality of the imposing government. For advocates, forced feeding is a civilised response to overcome the prisoners de‐civilising, irrational or insane actions. Alternatively, opponents argue force‐feeding is a form of violent torture that undermines notions of being civilised and as such is a challenge to the established self‐evaluation around morality, ethics, behaviour and notions of freedom and justice. The extent to which force‐feeding is considered a civilising safeguard for life, or a barbaric form of torture is informed by the habitus of those making the judgement, the range and depth of mutual identifications.

Whatever justifications were provided for force‐feeding, one common outcome is that during imprisonment the balance of power and dependency (Wouters 2019) between the prisoners and governments further shifted towards the latter. Nevertheless, as Elias outlined, within the internal dynamics of the relationship less powerful groups and individuals have some power. In the case of the hunger strikers their power was shown in continued struggles against the imposition of force‐feeding and the ongoing refusal to accept food despite the state's determination to break the protests. Moreover, the differing power dynamics between governments and opponents of force‐feeding was shown in how the practice was stopped in the UK while continuing in the USA. That force‐feeding varied within the same UK locations highlights how the method was influenced by medical practitioners' habitus and levels of mutual identification that incorporated or excluded the outsider prisoners within hierarchies of integration.

For instance, treatment of many suffragettes was informed by their shared or heightened status and dispositional gender expectations held by medical and legal practitioners. On the matter of suffrage and accompanying actions, especially when these escalated, the suffragettes were outsiders. In other ways they were connected to the patriarchal established and their associated roles as mothers, wives, sisters and daughters interwoven with male interdependencies across the socio‐economic spectrum. Within these figurations, the women also shared the purpose of Empire and sense of overarching civilisational superiority which shaped their treatment and the government's refusal to allow suffragettes to die. Yet these interconnections were not universal. The women were campaigning for the right to vote that was also denied to propertyless males. This challenge seems to have contributed to the disruption of suffragette rallies and the coarser treatment by male prison wardens, who tended to be part of the disenfranchised masses, whose status within patriarchal arrangements and hierarchies of integration were being threatened by universal suffrage. Wouters (2007) explained how more open, fluid forms of integration provide both greater opportunities for wider forms of we‐identification and insecurities and uncertainties for those within we‐groups that begin to disintegrate. Members of We‐groups experiencing a breakdown of social cohesion and solidarity can become vocal critics of these broader integrative figurations and have included men facing the threat of universal suffrage, gender employment and equal rights.

By comparison, most prearrest Irish republicans' jobs and lifestyles were weakly integrated into the figurations of the legal and medical professions. When a minority of republicans did share similar professional backgrounds such as lawyers and medical doctors, they had different levels of higher integration with Irish identification at odds with the prison and medical establishment's sense of being British. Consequently, the nature of republican actions was not usually offset by mutual identifications with officials, and they experienced less empathetic treatment. Nevertheless, mutual identification between some hunger strikers and prison wardens was on occasion tighter with common experiences around housing and social facilities. For instance, the different attitudes of wardens reported in the case of Frank Stagg and improved relationships, when force‐feeding was stopped, are indicative of changing relationships and levels of identification. Some wardens showed greater empathy while others were much more aggressive. The latter examples are indicative of weaker levels of integration with the prisoners, informed by a greater sense of threat by the rising Irish challenge to their self and national senses of status and common history.

Unlike when the suffragettes were no longer being imprisoned, the initial cessation of force‐feeding of republicans was driven by the protests that followed the death of Thomas Ashe and then in the longer term after gender stereotyping the Price sisters intersected with the death of Michael Gaughan. These events were part of wider shifts towards heightened sensitivities, more robust and deeper processes of pacification and empathy allied to the medical model becoming less dominant within the movement towards processes of rehabilitation and the protection of human rights.

The international shift towards the sensitisation of force‐feeding has yet to permeate the USA. There are a number of potential reasons for continuing practices within American institutions. When introduced in Guantanamo, the USA had declared itself at war with the notional and amorphous enemy of ‘Terror’. Fears and insecurities had heightened to a level that exceeded those experienced in the UK when facing smaller, more easily identifiable threats from suffragettes and Irish republicans. During the American time of crisis and heightened emotions there was widespread support for greater management of risks, enhanced social‐restraints and underpinning forms of behaviour that other parts in the Global North had been considering with increasing disdain. In other words, the normative code surrounding security of the nation‐state was given precedence over the freedom of others' activities and liberty which included the denial of the prisoners' freedom to die. During such shifts, the tensions between Western civilising normative codes identified by Elias (1996) from colonialism onwards, become more evident (Linklater 2021).

Treatment of prisoners, including force‐feeding, within Guantanamo and limited American protests, have also been informed by the distinct established We identity based on nationalism (Elias and Scotson 1965; Mennell 2007; Wouters 2007) which, when intersected with religion, restricted circles of identification to the exclusion of Muslim ‘outsiders’. This deep rooted American established habitus is formulated and dispersed within hierarchies of nationalism and religion which reinforces their sense of (white Christian) superiority and provide the basis for their claim for higher status (Mennell 2007). And on the reverse is the exclusionary criteria of difference on which to stigmatise the prisoners. With contemporaneous narrative interweaving with deep rooted American historical projections of morality, the medical lifesaving projection of forced feeding proved more powerful than marginalised objections to the barbarity of the methods.

In short, positioning forced feeding within ambiguities surrounding notions of civilised behaviour highlights characteristics associated with processes of de‐civilising such as weaker forms of mutual identification, greater imposition of social‐constraints, reduced freedoms and directed forms of violence within public institutions (Elias 1996). Within the UK, these contradictions proved too challenging to the national self‐consciousness and led to the ending of forced feeding. In America, the different history, more aggressive habitus and projected and exaggerated established characteristics intersect with fantasy levels of knowledge about danger, protection and the outsiders. The outcome is that physical, social and psychological impacts of force‐feeding are being restricted to hunger strikers and their restricted figurations with little or no effect on the dominant American sense of their civilised superiority.

## Conflicts of Interest

The authors declare no conflicts of interest.

## Data Availability

Data sharing is not applicable to this article as no new data were created or analysed in this study.
